# Bone Metastasis Challenge: New Ideas and Future

**DOI:** 10.3390/ijms24076161

**Published:** 2023-03-24

**Authors:** Manuel Scimeca

**Affiliations:** 1 Department of Experimental Medicine, TOR, University of Rome Tor Vergata, 00133 Rome, Italy; manuel.scimeca@uniroma2.it; 2 San Raffaele Roma Open University, Via di Val Cannuta 247, 00166 Rome, Italy; 3 UniCamillus, Saint Camillus International University of Health Sciences, 00131 Rome, Italy

Bone metastasis is a complex and challenging clinical problem, affecting patients with advanced stages of cancer [1]. According to epidemiological data, bone metastases occur in up to 70% of patients with advanced breast and prostate cancer, and up to 30% of patients with lung, kidney, and thyroid cancers [2]. The presence of bone metastases significantly reduces the quality of life of patients, causing severe pain, pathological fractures, and spinal cord compression [1].

In this scenario, the Special Issue entitled “Bone Metastasis Challenge: New Ideas and Future” of the *International Journal of Molecular Sciences* includes a total of six contributions: five original articles and one review providing significant advances in the understanding and treatment of bone metastasis.

The study by Mbese and Aderibigbe reported the potential of bisphosphonate-based conjugates and derivatives as therapeutic agents for osteoporosis, bone cancer, and metastatic bone cancer [3]. The authors highlight the ability of these agents to inhibit osteoclast activity, reduce bone resorption, and prevent the formation of new bone metastases. They also discuss the challenges associated with the use of these agents, including the potential for renal toxicity and the limited bioavailability of some derivatives. Overall, the study suggests that bisphosphonate-based conjugates and derivatives hold promise as therapeutic agents for bone-related diseases, but further research is needed to optimize their clinical efficacy and safety.

The additive benefits of the combination of Radium-223 Dichloride and Bortezomib in a systemic multiple myeloma mouse model have been investigated by Suominen and colleagues [4]. The results showed that this combination therapy not only inhibited the growth of multiple myeloma cells but also increased bone density and prevented bone destruction.

Furthermore, the osteoprotective effects of Loganic Acid on osteoblastic and osteoclastic cells and osteoporosis-induced mice were studied [5]. Experimental data suggest that loganic acid can promote osteoblast differentiation, inhibit osteoclast differentiation, and increase bone mineral density, which could be a promising treatment for osteoporosis and bone metastases. This study also found that the loganic acid treatment increased the expression of osteogenic markers and reduced the expression of bone resorption markers in osteoblastic and osteoclastic cells. In the in vivo investigations, authors demonstrated that the loganic acid treatment prevented bone loss and improved bone microarchitecture in osteoporotic mice.

In addition, a research paper investigated the expression profile of new marker genes involved in the differentiation of canine adipose-derived stem cells into osteoblasts [6]. This study provides important insights into the molecular mechanisms underlying osteoblast differentiation, which could lead to the development of novel therapies for bone regeneration and repair.

Cold atmospheric plasma (CAP) has also been investigated for its potential in promoting regeneration-associated cell functions of murine cementoblasts in vitro [7]. The results showed that CAP treatment significantly increased the proliferation, migration, and differentiation of cementoblasts, thus suggesting its potential role in bone regeneration.

Finally, the study of Panahipouret al. [8] investigated the role of TGF-β in the secretome of irradiated peripheral blood mononuclear cells in supporting in vitro osteoclastogenesis. The findings suggest that TGF-β may play an important role in the regulation of bone remodeling and the development of bone metastases.

In conclusion, studies published in the Special Issue “Bone Metastasis Challenge: New Ideas and Future” provide valuable insights into the molecular mechanisms underlying the development and progression of bone metastases and offer promising strategies for the prevention and treatment of this devastating disease. Moreover, the combination of molecular, pathological, and imaging data could open new and intriguing perspectives for developing therapies based on personalized medicine principles [9].
